# Organic Optocoupler with Simple Construction as an Effective Linear Current Transceiver

**DOI:** 10.3390/ma18010152

**Published:** 2025-01-02

**Authors:** Jaroslaw Jung, Arkadiusz Selerowicz, Jacek Ulanski, Ruslana Udovytska, Beata Luszczynska, Artur Zawadzki, Andrzej Rybak

**Affiliations:** 1 Department of Molecular Physics, Faculty of Chemistry, Lodz University of Technology, 116 Zeromskiego Str., 90-924 Lodz, Poland; arkadiusz.selerowicz@dokt.p.lodz.pl (A.S.); jacek.ulanski@p.lodz.pl (J.U.); ruslana.udovytska@p.lodz.pl (R.U.); beata.luszczynska@p.lodz.pl (B.L.); 2 ABB Corporate Technology Center, 13A Starowislna Str., 31-038 Krakow, Poland; artur.zawadzki@pl.abb.com (A.Z.); andrzej.rybak@pl.abb.com (A.R.)

**Keywords:** organic optocoupler, short circuited organic photodetector, DC and AC properties

## Abstract

In this study, it is shown that an efficient organic optocoupler (OPC) can be fabricated using commercially available and solution-processable organic semiconductors. The transmitter is a single-active-layer organic light-emitting diode (OLED) made from a well-known polyparavinylene derivative, Super Yellow. The receiver is an organic light-emitting diode (OLSD) with a single active layer consisting of a mixture of the polymer donor PTB7-Th and the low-molecular-weight acceptor ITIC; the receiver operates without an applied reverse voltage. OLED and OLSD have the same geometry and simple structure without any interlayers: glass/ITO/PEDOT:PSS/(active layer)/Ca/Al; the OPC is formed by OLED and OLSD which adhere tightly to each other. Despite its simple structure, the OPC showed a current transfer ratio of 0.13%, good linearity, and good dynamic performance: a three-decibel cutoff frequency of 170 kHz and response times to a step change in current at the OPC input of 2 μs. Compared to most organic OPC devices with similar performance parameters, where the transmitter and receiver have complex structures with additional interlayers between the active layers and electrodes and the need to apply a reverse voltage to the receiver, the simple design of our OPC reduces the number of fabrication steps and greatly simplifies the device fabrication process.

## 1. Introduction

An optocoupler is a device that can be used to transmit alternating signals between galvanically separated electrical circuits [1,2]. In real organic optocouplers (OPCs), the ability to transmit an AC signal without distortion is limited [3,4]. These limitations result from external conditions (in the case of hermetically encapsulated optocouplers, it is mainly the operating temperature) and the materials and manufacturing technology used [5,6,7,8,9,10]. These limitations include the three-decibel cutoff frequency ($f_{-3dB,OPC}$) for the highest harmonic of the alternative current transmitted to the receiver without amplitude attenuation and the current transfer coefficient (CTR), which indicates the efficiency with which the optocoupler converts the current applied to the input into the current detected at the output.

It should be noted that even with a transmitter and receiver with very good operating parameters, it is not always possible to design an optocoupler that can transmit AC signals with low nonlinear distortion. To obtain an effective device, transmitters and receivers should be selected so that their light emission and absorption spectra and linear operating ranges overlap partially [11] or completely [12,13]. In addition, additional interlayers are often used in photodiodes to prevent current leakage when reverse bias is applied [14,15,16,17]. However, it is possible to use simpler photodetector designs and achieve good results, as we demonstrate in this paper.

**Figure 1:**
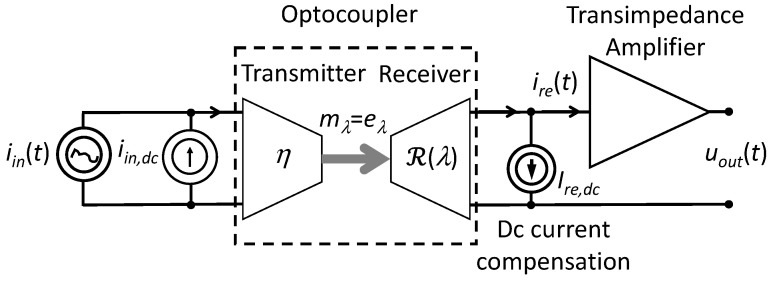
Schematic diagram of an optocoupler with dc $i_{in,dc}$ and ac $i_{in}\left(t\right)$ input current sources and a current source $i_{re,dc}$ designed to eliminate the DC component from the current at the output of the optocoupler.

Organic optocouplers are constructed using an organic light-emitting diode (OLED) as the transmitter and an organic light-sensitive diode (OLSD) as the receiver. OLSD devices can operate as photodetectors with short-circuited electrodes. The active layers in the OLED and OLSD are made of organic semiconductors that can be processed by solution methods, making them easy to manufacture. Encapsulated OPCs exhibit relatively good long-term stability and can transmit current signals in a wide frequency bandwidth while maintaining good linearity [18,19,20,21]. A diagram of an optocoupler with a photodetector with short-circuited electrodes in the receiver circuit is shown in Figure 1.

In order to obtain the least distorted signals at the output of the optocoupler, it is necessary that in the frequency range corresponding to the frequency band of the transmitted signal, the current recorded at the output ($i_{re}$) is proportional to the current supplied to the input ($i_{in}$). For this purpose, a direct current ($i_{in,dc}$), is supplied to the input of the device, so that the device works in the linear range of the current transfer characteristic. An alternating current ($i_{in}\left(t\right)$), by means of which the signal is transmitted, is added to the constant component of current $i_{in,dc}$. The transmitter (i.e., OLED) emits alternating light with spectral emittance $m_{\lambda }$ (where *λ* is the light wavelength) proportional to the variable component of current $i_{in}\left(t\right)$. The receiver (i.e., OLSD), exposed to radiation, converts the light energy into current $i_{ph}$. The output circuit contains a current source for eliminating the DC component of the receiver current ($i_{re,dc}$). The transimpedance amplifier (serving as an ideal ammeter with zero internal resistance) connected to the correction circuits converts the AC component of the receiver current ($i_{re}\left(t\right)$) into voltage ($u_{out}\left(t\right)$).

**Figure 2:**
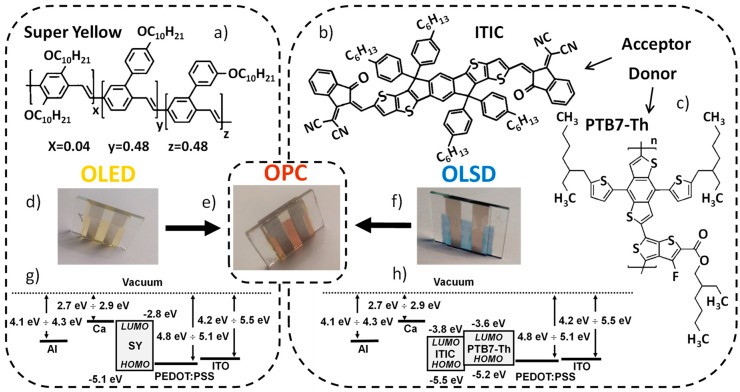
Materials and devices. Organic semiconductors used to produce active layer of (**a**) OLED (Super Yellow) and of OLSD ((**b**) acceptor ITIC and (**c**) donor PTB7-Th); photos of (**d**) OLED, (**e**) OPC, and (**f**) OLSD; (**g**,**h**) work functions for electrodes (Al, ITO), hole-transporting layer (PEDOT:PSS), and electron-transporting layer (Cu) and LUMO and HOMO energies of semiconductors [35,36].

This paper presents an organic optocoupler with a simple structure consisting of the combination of an OLED as a transmitter and an OLSD as a receiver, wherein the active layers in the OLED and OLSD are fabricated by solution methods from well-known and widely studied organic semiconductors. The advantage of the device is the single-layer structure of both the OLED and OLSD, without interlayers. It is also important that, unlike most known organic optocouplers [9,22,23], the devices described in this paper do not require a reverse voltage on the receiver side. As the active layer in the OLED, a poly(para-phenylenevinylene) copolymer with the trade name SuperYellow (SY) (Figure 2a) [24,25,26,27,28] was used. SY is a well-known material that emits light in a yellow color and transports both holes and electrons. This material allows for the construction of OLEDs with a single active layer, which greatly simplifies the device fabrication process compared to most OLED devices with a complicated multi-layer structure. The active layer of the OLSD was a mixture of a donor and acceptor forming a bulk heterojunction. The donor was the polymer poly([2,6′-4,8-di(5-ethylhexylthienyl)benzo [1,2-b;3,3-b]dithiophene]{3-fluoro-2[(2-ethylhexyl)carbonyl]thieno [3,4-b]thiophenediyl}) with the commercial name PTB7-Th [29,30,31] (Figure 2c), and the acceptor was the low-molecular-weight, non-fullerene compound 3,9-bis(2-methylene-(3-(1,1-dicyanomethylene)-indanone))-5,5,11,11-tetrakis(4-hexylphenyl)-dithieno [2,3-d:2′,3′-d’]-s-indaceno [1,2-b:5,6-b’]dithiophene with the abbreviation ITIC [14,32,33,34] (Figure 2b). The OLED and OLSD had the same architecture, ITO/PEDOT:PSS/(active layer)/Ca/Al, and the same electrode geometry and were separated only by thin glass plates, i.e., glass supports of OLED and OLSD. It was therefore assumed that the spectral emittance of the emitter $\left(m_{\lambda }\right)$ was equal to the spectral irradiance ($e_{\lambda }$) of the receiver.

Optocouplers manufactured in this way have good performance parameters, such as a high current transfer ratio (CTR) of 0.13%, a wide frequency band (three-decibel cutoff frequency of 170 kHz), and good linearity.

## 2. Materials and Methods

### 2.1. Sample Preparation and Characterization

Optocouplers were fabricated in two ways. One was to produce OLED devices (Figure 2d) and OLSD devices (Figure 2f) separately on glass substrates with six indium tin oxide (ITO) electrodes (anodes) (purchased from Ossila, S101, Sheffield, UK) and then bond the two devices closely together. The second method was to fabricate an optocoupler with ITO electrodes pre-patterned on both sides of the glass substrate (made by Instytut Fotonowy, Kraków, Poland) (Figure 2e). The geometry and area of the electrodes (*S* = 4.5 × 10^−6^ m^2^) for both one-sided and two-sided covered glass were identical. Substrates were ultrasonically washed twice in acetone and then twice in isopropanol for 5 min each time. Then, they were dried in a stream of nitrogen and then exposed to oxygen plasma for 150 s using a plasma surface treatment machine with a power of 200 W (Diener, Atto, Ebhausen, Germany).

The hole transport layers were prepared from a commercial PEDOT:PSS solution (purchased from Ossila, Al 4083) by spin coating them on ITO plates at a speed of 3000 rpm for 30 s. The substrates were then heated at 120 °C for 10 min in a nitrogen atmosphere. The active layer of the luminescent diodes was made by spin-coating (2000 rpm for 30 s) from a solution of Super Yellow (Sigma-Aldrich, St. Louis, MO, USA) in toluene (5 mg of polymer in 1 mL of solvent). The active layer of the OLSD was deposited from a mixture of ITIC and PTB7-Th in a mass ratio of 1:1. The total concentration of the solution was 20 mg of ITIC:PTB7-Th mixture per 1 mL of chloroform. Similar to OLED, the active layer of the OLSD device was prepared by spin coating (2500 rpm, 60 s). A scheme of the layer structure of the optocoupler is presented in Figure S1 in Supplementary Information.

The thicknesses of the prepared layers, measured using a Bruker DEKTAK XT needle profilometer (Billerica, MA, USA), were as follows: PEDOT:PSS-30 nm, SY-70 nm, and ITIC:PTB7-Th-150 nm. Finally, the metallic shared cathode was vacuum-evaporated on all six devices. This electrode was composed of a 10 nm layer of calcium evaporated directly on the surface of the active layer and a 100 nm thick protective layer of aluminum. The samples were prepared in a glovebox system with a controlled nitrogen atmosphere and then encapsulated to protect the organic layers from oxygen and moisture using an adhesive, non-destructive matrix encapsulation epoxy (Ossila, E132). Curing of epoxy resin was achieved by UV illumination at a wavelength of about 350 nm for 2 min. The resin in conjunction with a glass coverslip provided a robust barrier, ensuring an extended service life during the measurement and storage of samples.

### 2.2. Optical and Electrical Properties of Devices

DC Characterization

The DC current–voltage characteristics of OLSD and OLED were collected using a Keithley 2400 source measurement unit. The photocurrent was measured under OLSD illumination with the use of a solar simulator (AM1.5, 1000 Wm^−2^, LOT Oriel, LS 0110-1000). The density of radiant flux per unit area of OLED were controlled with homemade equipment consisting of an Ocean Optics spectrometer (365–900 nm), the Keithley 2400 source-meter with a computer, and appropriate software. The external quantum efficiency (*EQE*) spectra for OPDs were determined using a photoelectric spectrometer (Instytut Fotonowy). The OLEDs were powered by a Matrix MPS-3005L-3 DC power supply and the current was measured with the use of a Fluke 8808A multimeter. The bias voltage applied to the OLSD and the photocurrent control were performed using a Keithley 2400 source meter.

AC Characterization

The OLEDs were powered by the sum of the DC and AC voltage from the sinusoidal- or square-wave generator (Matrix 8216A). The DC component of the bias voltage determined the operating conditions of the OLED devices (the operating point on the current–voltage characteristics). The AC analysis of the photocurrent was performed by illuminating the OLSD with an inorganic white light-emitting diode. Two Rigol DS1052E l oscilloscopes, electrically isolated from each other, were used to measure the dynamics of the signal changes. One was used to measure the LED or OLED supply voltage and the other to monitor the output voltage of the high-speed transimpedance amplifier (Femto, DHP-CA-100) that converts the OLSD photocurrent into voltage. The output voltage of the transimpedance amplifier was proportional to the measured current over the full range of the frequency spectrum. A bias voltage applied to the photodiode was supplied by the voltage source (Matrix MPS-3005L-3). A silicon photodiode with a dedicated amplifier (ThorLabs, PDA36A-EC) was used to analyze the changes in the light emitted by the OLED over time. A scheme of the DC and AC control systems is presented in Figure S2 in the Supplementary Information.

## 3. Results and Discussion

**Figure 3:**
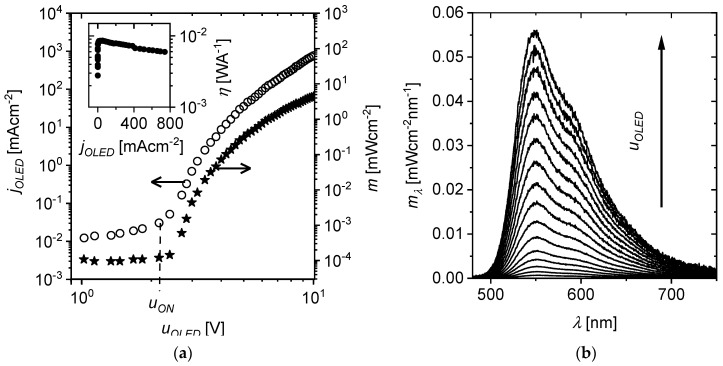
(**a**) Current–voltage characteristics $j_{OLED}\left(u_{OLED}\right)$ (circles) and the dependence of the total radiant emittance on the voltage $m\left(u_{OLED}\right)$ (stars) for OLED based on SY; the symbol $u_{ON}$ denotes the threshold voltage; the inset shows the dependence of the current efficiency η on the current density $j_{OLED}$; (**b**) spectral emittance $m_{\lambda }$ for different voltage values $u_{OLED}$.

An example of the current–voltage characteristics of an OLED based on SY is shown in Figure 3a. For a supply voltage ($u_{OLED}$) lower than the turn-on voltage $u_{ON}\;$= 2.1 V, a current flows in the diode, for which the density ($j_{OLED}$) is a linear function of the voltage. When the turn-on voltage is exceeded, the current density suddenly increases. According to the literature [36,37], the value of $u_{ON}$ corresponds to the voltage of the built-in field, which, after multiplying by the elementary charge (*q*), is equal to the energy difference between the work functions of electrons exiting the anode and cathode (made of PEDOT:PSS and calcium layers, respectively). For voltages above this, light emission occurs.

### 3.1. Characteristics of the OLED Emitter

In optocouplers, the detector is an electronic device and not the human eye, so instead of photometric quantities (such as luminance), spectroscopic quantities such as the spectral density of radiation emittance $m_{\lambda }$ and radiation emittance *m* were used to describe the operation of the OLED transmitter properties. The spectra $m_{\lambda }$ measured for different values of voltage $u_{OLED}$ are shown in Figure 3b. The dependence of the total radiant emittance on voltage $m\left(j_{OLED}\right)$ shown in Figure 3a was determined from the spectral emittance $m_{\lambda }\left(j_{OLED}\right)$ measured using Equation (1):

(1) $$m\left(j_{OLED}\right)=\int_{0}^{\infty }m_{\lambda }\left(j_{OLED}\right)d\lambda$$

Based on the *I-V* characteristic and the $m\left(u_{OLED}\right)$ dependence, current efficiency was determined as a function of current density ($\eta \left(j_{OLED}\right)=m\left(j_{OLED}\right)/j_{OLED}$). The graph in the inset of Figure 3a shows that as current density increases from 0.1 to 800 mA/cm, current efficiency slowly decreases from 85 to 60 A/W.

An important parameter of the transmitter is the speed at which the OLED emits light after the voltage is applied. To test the effectiveness of the emission of alternating light, the OLED devices were supplied with a voltage containing a constant time component, $u_{OLED,dc}$, and a harmonic variable component: $u_{OLED\sim }\left(t\right)=u_{0,OLED}\sin\left(2\pi ft\right)$. For the value of the constant voltage component $u_{OLED,dc}$ = 7 V and the amplitude of the variable component $u_{0,OLED}$ = 0.1 V, the current density varied from 190 to 210 mAcm^−2^. The operating point of the OLED was the current density $j_{OLED,dc}\;$= 200 mAcm^−2^ and the corresponding radiation emittance $m_{dc}$ = 16 Wm^−2^.

Based on the series of measured oscillograms for different frequencies, the relative amplitude of the radiation emittance, $m_{0,rel}\left(f\right),$ expressed in decibels, was calculated:

(2) $$m_{0,rel}\left(f\right)=20log\left[m_{0}\left(f\right)/m_{0}\left(10Hz\right)\right]$$

where $m_{0}\left(f\right)$ is the amplitude of the alternating component of the radiation emittance. Then, the amplitude spectrum of the radiation emittance was prepared and on its basis, the three-decibel cutoff frequency, $f_{-3dB,OLED}$, was determined to be $f_{-3dB,OLED}$ ≈ 640 kHz.

The speed of OLED devices was limited by contact resistance, $R_{s,OLED}$, differential resistance of the OLED, $R_{diff,OLED}$ (equal to the inverse derivative of the I-V characteristic for a fixed voltage value), and geometric capacitance $C_{OLED}=S\varepsilon \varepsilon_{0}/L$ (where $\varepsilon {,\;\varepsilon }_{0},\;S,$ and *L* are the vacuum permittivity, the relative permittivity of SY, the OLED area, and the SY layer thickness, respectively).

From the I-V characteristic presented in Figure 3a, it follows that for a voltage of 7 V, resistance $R_{diff,OLED}$ was equal to 220 Ω, and based on the electrical equivalent model of OLED (made on the basis of impedance tests, described in [36]), contact resistance $R_{s,OLED}=100\;\Omega$ was calculated. The three-decibel cutoff frequency is

(3) $$f_{-3dB,OLED}=1/\left(2\pi \tau_{RC,OLED}\right)$$

where $\tau_{RC,OLED}=2\pi C_{OLED}R_{diff,OLED}R_{s,OLED}/\left(R_{diff,OLED}{+R}_{s,OLED}\right)$ is the time constant for the simplified OLED equivalent circuit shown in Figure S3 in the Supplementary Information. Based on the above formula and the experimentally obtained results, the value of the dielectric constant of SY, ε, was calculated from the following formula:

(4) $$\varepsilon =\frac{L\left(R_{diff,OLED}{+R}_{s,OLED}\right)}{2\pi f_{-3dB,OLED}S\varepsilon_{0}R_{diff,OLED}R_{s,OLED}}$$

The estimated ε value was 6.3.

### 3.2. Characteristics of the OLSD Photodetectors

**Figure 4:**
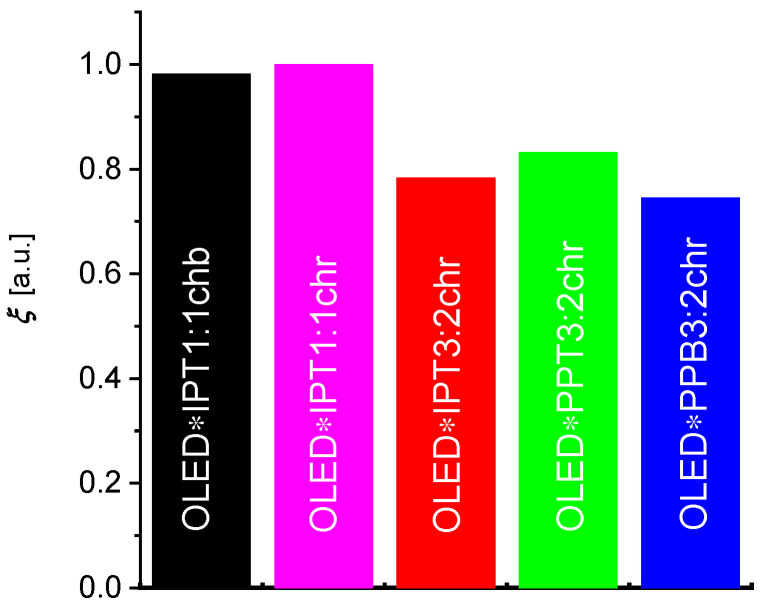
Comparison of relative overlap integrals *ξ* of spectral emittance $m_{\lambda }$ of OLED based on SY transmitter and responsivity $R\left(\lambda \right)$ of OLSD photodetectors based on different donor–acceptor mixtures.

Five different OLSD photodetectors were tested as a receiver in optocouplers with the OLED transmitter based on SY. It was assumed that the optocouplers should fulfill three basic conditions: (a) they should transmit signals from the transmitter to the photodetector with high efficiency, (b) they should be characterized by a high signal transmission rate while maintaining good DC parameters, and (c) they should not lose their electro-optical properties after encapsulation and operate stably under normal atmospheric conditions for at least several weeks. The characteristics of the tested OLSD devices with active layers made of mixtures of different semiconductors are given in the Supplementary Information (Figure S4 and Table S1). Based on the measured spectra of emittance spectral density m_λ for the OLED emitter (measured for $j_{OLED}$ = 0.11 mAcm^−2^) and the OLSD response $R\left(\lambda \right)$, the overlap integrals were calculated. These were used to determine the ability of each transmitter/receiver pair to effectively convert light energy into photocurrent. The highest values of the relative overlap integral *ξ* (see Figure 4) were found for the OLED/IPT1:1chb and OLED/IPT1:1chf systems.

(5) $$\xi =\frac{\int_{300\;\mathrm{nm}}^{800\;\mathrm{nm}}m_{\lambda }R\left(\lambda \right)d\lambda }{\int_{300\;\mathrm{nm}}^{800\;\mathrm{nm}}m_{\lambda }R_{IPT1:1chr}\left(\lambda \right)d\lambda }$$

The semiconductors that make up the active layer of OLSD devices are unstable in air, so OLSD devices were fabricated and characterized in glove boxes filled with nitrogen. The IPT1:1chb system was found to have the best stability in air after encapsulation of the OLSD devices tested. It was also found that this OLSD converted light signals to photocurrent at a much higher rate than the other OLSD devices and showed good DC properties without an applied reverse voltage. Therefore, this system was selected for use in the optocoupler together with SY-based OLED.

### 3.3. Characteristics of the Optocoupler

**Figure 5:**
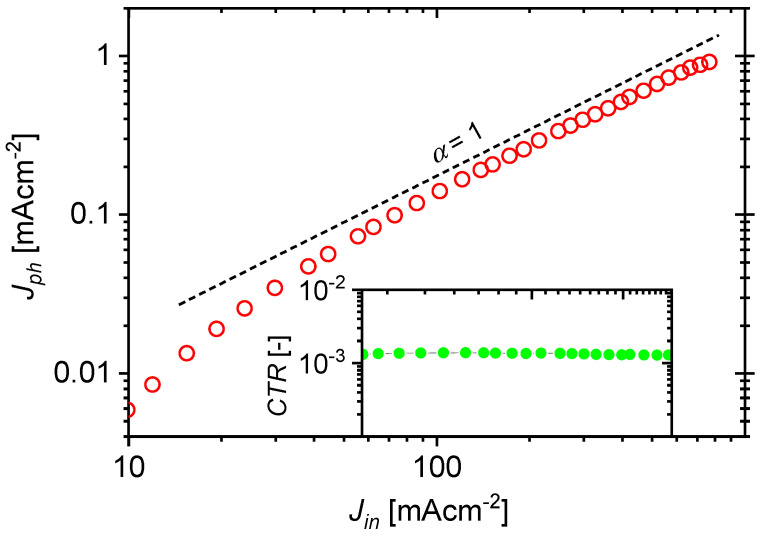
Dependence of photocurrent density at photodetector output $j_{ph}$ on current density at transmitter input $j_{in}$ for the OLED/OLSD optocoupler. The inset shows the dependence of current ratio *CTR* on $j_{in}$ in the linear range of the optocoupler operation.

Constant current measurements were performed on the constructed OLED/OLSD devices and the dependence of the photocurrent density on input current $j_{ph,dc}\left(j_{in,dc}\right)$ was determined. The current generated by light in the receiver, $j_{re}$, was measured using a transimpedance amplifier without reverse-biasing the OLSD photodetector. It was assumed that the current density in the circuit at the receiver output was equal to the photocurrent density $j_{ph}$ in OLSD; according to the optocoupler model shown in Figure 1, $j_{re}=j_{ph}R_{sh,re}/\left(R_{sh,re}+R_{s,re}\right)$, which for $R_{sh,re}\gg R_{s,re}$ gives $j_{re}≅j_{ph}$. Figure 5 shows the measured current characteristic for an example optocoupler. The characteristic is linear for the current density at the OPC input in the range from 55 to 580 mAcm^−2^.

The key parameter that characterizes an optocoupler is the current transfer ratio (*CTR*), which is defined as the ratio of the current detected at the receiver output to the current supplied to the transmitter input:

(6) $$\begin{array}{c} CTR=\frac{i_{re}}{i_{in}}\approx \end{array}\frac{i_{ph}}{i_{in}}=\frac{j_{ph}}{j_{in}}\frac{S_{ph}}{S_{em}}$$

where $S_{em}$ and $S_{ph}$ are the surfaces of the emitter and photodetector, respectively (for the tested OLED/OLSD, both surfaces were the same; $S_{ph}/S_{em}=1$).

An important requirement is that the range of variations in the current applied to the device input, for which the value of *CRT* coefficient remains constant, should be as wide as possible. For a current density range $j_{in}$ from 55 to 580 mAcm^−2^, where the dependence $j_{ph,dc}\left(j_{in,dc}\right)$ could be considered linear, the *CRT* value was about 0.13% and was similar to those found in the literature [4,8,38]. However, in the tested systems, the *CTR* value was only approximately constant over a limited range of current density $j_{in,dc}$. Moreover, the current transfer ratios for the DC and AC components, ${CTR}_{dc}=i_{ph,dc}/i_{in,dc}$ and ${CTR}_{ac}=i_{ph,rms}/i_{in,rms}$, respectively (where the rms subscript denotes the root mean square value), were not equal for all current density values.

For time-varying signals, for frequencies above the three-decibel optocoupler cutoff frequency ($f_{-3dB,OPC}$), ${CTR}_{ac}$ decreased with increasing *f* according to the formula

(7) $$\begin{array}{c} \;{CTR}_{ac}\left(f\right)={CTR}_{0}{\left(1+\frac{f^{2}}{f_{-3dB,SY}^{2}}\right)}^{-1/2} \end{array}$$

where ${CTR}_{0}$ is the current transfer ratio for $f\ll f_{-3dB,OPC}$.

**Figure 6:**
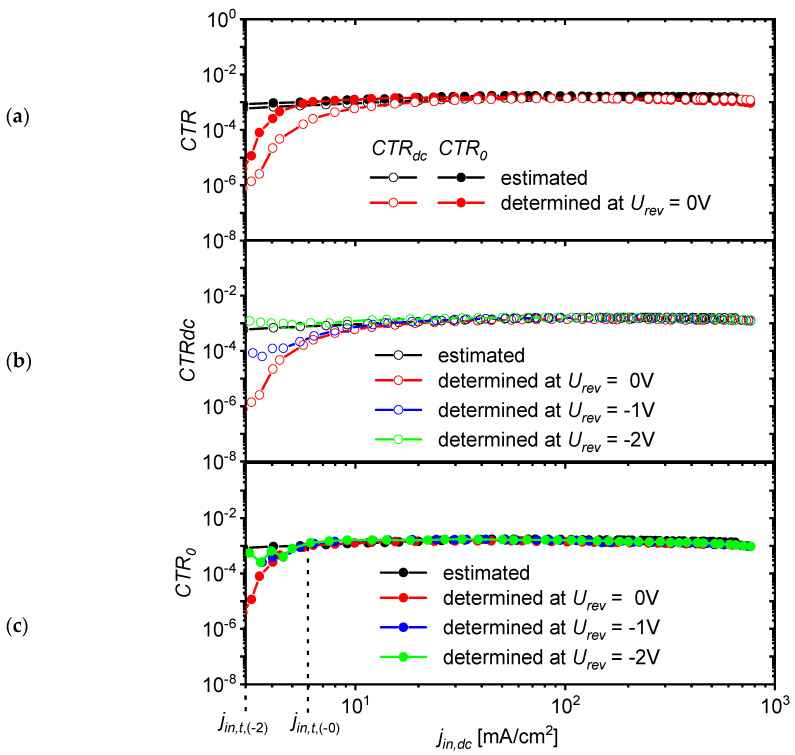
Comparison of the variations in direct ${CTR}_{dc}$ and alternating ${CTR}_{0}$ current transfer ratios as a function of the density of direct current component $j_{in,dc}$ for (**a**) the experimentally determined DC and AC current transfer ratios for the OLED/OLSD optocoupler at reverse voltage $u_{rev}\;$= 0 and their equivalents estimated based on the experimental results obtained for the OLED transmitter and the OLSD photodetector; (**b**) direct ${CTR}_{dc}$, and (**c**) alternating ${CTR}_{0}$ current transfer ratios determined for the photodetector biased with the reverse voltage $u_{rev}$ of two different values.

In Figure 6a, it is visible that the experimentally determined ${CTR}_{dc}$ and ${CTR}_{0}$ values decrease with current density $j_{in}$ below a certain threshold value of current density $j_{in,t}$. This threshold value $j_{in,t}$ could be reduced by biasing the OLSD photodetector with a reverse voltage ($u_{rev}$). In order to check what effect the reverse voltage polarization of the OLSD photodetector may have on the linearity of current transfer, the characteristics $j_{ph,dc}\left(j_{in,dc}\right)$ were measured for $u_{rev}=-1\;V$ and $u_{rev}=-2\;V$. The photocurrent density $j_{ph,dc}$ was calculated as the difference between receiver current density ($j_{re,dc}$) for $j_{in,dc}>0\;A$ and the leakage current ($j_{re,l}$) of the receiver at $j_{in,dc}=0\;A$ ($j_{ph,dc}=j_{re,dc}-j_{re,l}$).

To estimate the values of ${CTR}_{dc}$ and ${CTR}_{0}$, two approaches were used. In the first one, the coefficients were estimated based on independent tests performed separately for the receiver and transmitter (these coefficients are marked with the upper index *e*—estimated), and the second way was to determine these coefficients according to the definition as the ratio of the measured current at the OPC output and the current supplied to its input (these coefficients are marked with the upper index *d*—determined).

First, according to the definition of responsivity, the total photocurrent density was determined according to the formula $\begin{array}{c} j_{ph}=\int_{\lambda_{1}}^{\lambda_{2}}m_{\lambda }R\left(\lambda \right)d\lambda \end{array}$. The dependence of the spectral density of emittance of the transmitter radiation $m_{\lambda }$ on the current density at the transmitter input $j_{in,dc}=j_{OLED,dc}$ for OLED (Figure 3) and the responsivity spectrum ($R\left(\lambda \right)$) of the receiver) for OLSD were determined experimentally (see Supplementary Information Figure S4). Then, according to Equation (6), ${CTR}_{dc}^{\left(e\right)}$ was calculated. It was assumed that for small signals of the variable component, the approximate equation holds $j_{ph,rms}/j_{pin,rms}\begin{array}{c} \approx \end{array}dj_{ph,rms}/dj_{pin,rms}$. Based on this, ${CTR}_{0}^{\left(e\right)}$ was calculated as follows:

(8) $${CTR}_{0}^{\left(e\right)}\left(j_{in,dc}\right)=\int_{\lambda_{1}}^{\lambda_{2}}\frac{dm_{\lambda }\left(j_{in,dc}\right)}{dj_{in,dc}}R\left(\lambda \right)d\lambda$$

The determined dependencies ${CTR}_{dc}^{\left(e\right)}\left(j_{in,dc}\right)$ and ${CTR}_{0}^{\left(e\right)}\left(j_{in,dc}\right)$ were treated as reference curves for coefficients ${CTR}_{dc}^{\left(d\right)}\left(j_{in,dc}\right)=j_{ph,dc}\left(j_{in,dc}\right)/j_{in,dc}$ and ${CTR}_{0}^{\left(d\right)}\left(j_{in,dc}\right)=dj_{ph,dc}\left(j_{in,dc}\right)/dj_{in,dc}$ determined for the optocoupler created by assembling (i.e., simply by putting together) this transmitter–receiver pair.

Figure 6a compares the plots of the ${CTR}_{dc}\left(j_{in,dc}\right)$ and ${CTR}_{0}\left(j_{in,dc}\right)$ dependences determined by using the two methods described above for the case where the photodetector was not reverse-biased ($u_{rev}$ = 0 V). For a current density higher than 20 mAcm^−2^, all determined DC coefficients had very similar values in the range from 1.3 × 10^−3^ to 1.7 × 10^−3^. However, as the current density decreased below 20 mAcm^−2^, the coefficients ${CTR}_{dc}^{\left(d\right)}$ had lower values in relation to ${CTR}_{dc}^{\left(e\right)}$. A similar trend was observed for the AC coefficients, with significant differences between the estimated and experimentally determined ${CTR}_{0}$ values occurring only for the density of the direct current component $j_{in,dc}$ below 6 mAcm^−2^.

As shown in Figure 6b, the values of coefficients ${CTR}_{dc}^{\left(e\right)}$ and ${CTR}_{dc}^{\left(d\right)}$ have approximately similar values for a current density $j_{in,dc}$ higher than 15 mAcm^−2^, independently of the reverse voltage values applied to the OLSD photodetector. When the current density $j_{in,dc}$ is lower than 15 mAcm^−2^, the values of coefficients ${CTR}_{dc}^{\left(e\right)}$ and ${CTR}_{dc}^{\left(d\right)}$ are different. However, after applying the reverse voltage to the photodetector, these differences decrease; the higher the absolute value of the $\left|u_{rev}\right|$, the smaller this difference is. A similar effect of applying the reverse voltage to the photodetectors was observed for the AC coefficients ${CTR}_{0}$ (Figure 6c).

The discrepancy between the estimated and experimentally determined *CTR_dc_* and *CTR*_0_ coefficients could be due to the phenomenon of electron trapping in the acceptor phase or/and holes in the donor phase in the bulk heterojunction in OLSD. Trapping reduces the population of mobile charge carriers and therefore reduces the photocurrent intensity. For a given trap concentration, the trapping effect will be particularly pronounced for low photocurrents because a relatively large fraction of the photogenerated charge carriers can get stuck in the traps. After applying the reverse voltage $u_{rev}$ an additional external electric field is created in the heterojunction, which, according to the Poole–Frenkel mechanism, reduces the energy barrier for carriers to leave the traps [39,40]. At a sufficiently high voltage, the residence time of the carriers in the traps is significantly reduced, and the trapping of the charge carriers has little effect on the current flow.

**Figure 7:**
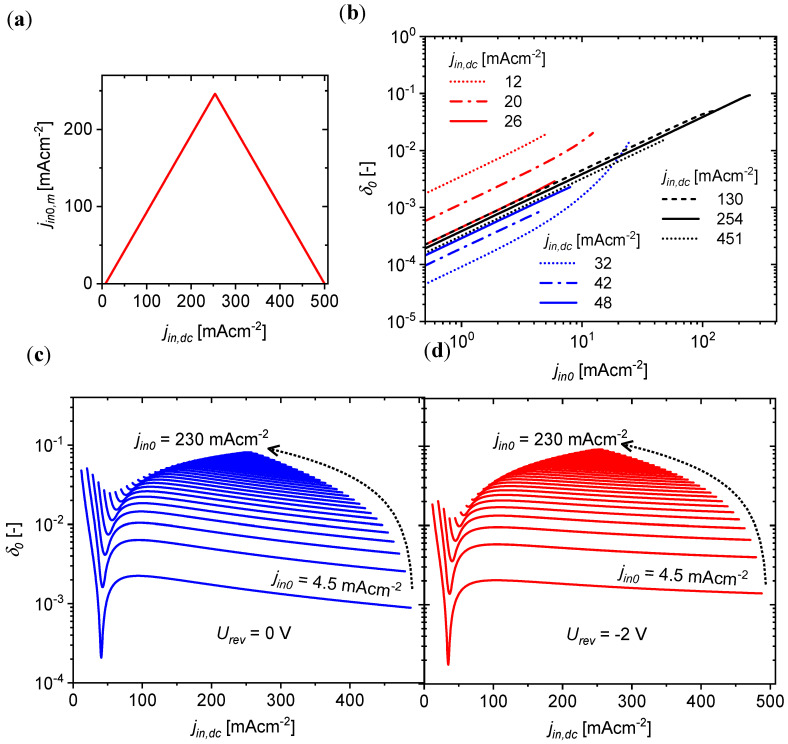
(**a**) The highest possible value of the amplitude of the variable component of current density $j_{in0,m}$ as a function of $j_{in,dc}$ for the assumed range of linear operation of OLED/OLSD for a photodetector unpolarized by reverse voltage; (**b**) dependence of coefficient $\delta_{0}$ on $j_{in,0}$ for different values of the constant component of current $j_{in,dc}$ in the range 12–451 mAcm^−2^; (**c**,**d**) coefficient $\delta_{0}$ as a function of the constant component of current density $j_{in,dc}$ supplied to the OPC input for different values of the amplitude of variable component $j_{in,0}$, for two cases: (**c**) a non-polarized photodetector and (**d**) a photodetector polarized with a reverse voltage $u_{rev}\;$= −2 V.

In the case where $f<f_{-3dB,OPC}$ and $j_{in}>j_{in,t}$, the DC and AC current transfer ratios have similar values, ${CTR}_{dc}\approx {CTR}_{0}$, which apparently do not depend on the current intensity, as can be seen in Figure 6. However, the DC and AC *CTR* values are not exactly equal and constant in the OPC input current density ranges described above. The CTR coefficients changed slightly with the change in current density $j_{in,dc},$ resulting in the deterioration of linearity of the transmitting of high-amplitude AC currents ($j_{in0}$). In order to estimate the degree of this nonlinearity, we assumed that the range of linear operation of the example OLED/OLSD optocoupler for $u_{rev}$ = 0 V was within the current density limits $j_{in,dc}$ from the threshold value $j_{in,t}$ = 6 mAcm^−2^ to the maximum value $j_{in,m}$ = 500 mAcm^−2^ (Figure 6c). For the assumed range of the constant component of current density, the maximum possible amplitude of the variable component of current density ($j_{in0,m}$) depended on the value of the constant component $j_{in,dc}$ and was $j_{in0,m}=\left(j_{in,dc}-j_{in,t}\right)/2$ for $j_{in,dc}\leq \left(j_{in,m}-j_{in,t}\right)/2$ or $j_{in0,m}=\left(j_{in,m}-j_{in,dc}\right)/2$ for $j_{in,dc}\geq \left(j_{in,m}+j_{in,t}\right)/2$, as shown in Figure 7a.

For $j_{in,dc}\in \left(j_{in,t},j_{in,m}\right)$, the coefficients ${CTR}_{0}$ changed slightly with the changing current, and their deviation from the mean value ($\bar{{CTR}_{o}}$) was expressed by coefficient $\delta_{0}$ defined as

(9) $$\delta_{0}\left(j_{in,dc},j_{in0}\right)=\frac{\sqrt{\int_{j_{in,dc}-j_{in0}}^{j_{in,dc}+j_{in0}}{\left({CTR}_{o}\left(\xi \right)-\bar{{CTR}_{o}}\left(j_{in,dc},j_{in0}\right)\right)}^{2}d\xi }}{\bar{{CTR}_{o}}\left(j_{in,dc},j_{in0}\right)}$$

where

(10) $$\bar{{CTR}_{o}}\left(j_{in,dc},j_{in0}\right)=\frac{1}{2j_{0}}\int_{j_{in,dc}-j_{in0}}^{j_{in,dc}+j_{in0}}{\left({CTR}_{o}\left(\xi \right)-{CTR}_{o}\left(j_{in,dc}\right)\right)}^{2}d\xi$$

The value of $\delta_{0}$ depended on the choice of the constant component of current density $j_{in,dc}$ (OPC operating point) and the amplitude of the variable component of signal $j_{in0}$. Figure 7b shows the dependencies of the $\delta_{0}$ coefficient on the amplitude of the variable signal for different values of the constant component of current $j_{in,dc}$ in the range 12–451 mAcm^−2^. In each case, regardless of the value of the constant component of the current adopted, the nonlinearity increased as the amplitude of the variable component $j_{in0,m}$ increased. This is intuitive, since one might expect that the smaller the signal amplitude relative to the DC component of the input current, the better the linear function that can be used to approximate the current characteristic of the optocoupler in the immediate vicinity of the operating point (Figure 5).

Plots of the dependence of the $\delta_{0}$ coefficient on the DC component of the current density for different values of the AC signal amplitude have been drawn for an optocoupler with an unpolarized photodetector ($u_{rev}$ = 0 V—Figure 7c) and a polarized one ($u_{rev}$ = −2 V—Figure 7d). After exceeding the current density $j_{in,dc}$ (e.g., 100 mAcm^−2^), which is characteristic for the amplitude $j_{in,0}$ (e.g., 4.5 mAcm^−2^), the $\delta_{0}$ coefficient decreased, which was caused by the slightly decreasing current efficiency *η* with increasing current characteristic of OLED devices (see the inset in Figure 3a). In the whole range of current densities tested at the OPC input, $\delta_{0}$ had a higher value for $u_{rev}$ = −2 V, which indicates a deterioration in the linearity of light conversion into photocurrent by the optocoupler after polarizing the receiver with a voltage.

To operate in the third quadrant of the I-V characteristic and to broaden the frequency band photodiodes working as the photodetectors in optocoupler require reverse polarization [7,41,42,43,44,45]. However, from the above analysis, it is clear that in order to minimalize linearity degradation, the photodetector should be polarized with the lowest possible reverse voltage or should not be polarized at all. In addition, the following rules must be followed: (a) small values of direct current from the range in which *δ*_0_ reaches the lowest value should be used for alternating signals with low amplitude, or (b) for higher-amplitude alternating signals, the highest possible values of direct current should be used. On the other hand, one should be aware that too small an amplitude of AC current and/or too large a DC component can hinder transmission. The limitation of a small AC amplitude is the noise occurring in the transmitter, receiver, and amplifier, as well as the fluctuations of the power of the emitted light. It is often necessary to use a preamplifier to increase the AC amplitude and improve the signal-to-noise ratio before feeding the signal to the OPC input. However, this may preclude the use of variant a), as small values of direct current are only suitable for small AC signal amplitudes (Figure 7c,d). In turn, the use of large values of direct current (variant b) will contribute to an increase in shot noise in the photodetector, which can also worsen the signal-to-noise ratio at the OPC output. In the case of polarization of the OLSD photodetector with reverse voltage, the leakage current can increase significantly with the increasing voltage $u_{rev}$ and then the detectivity [44,46,47] of the receiver is drastically worsened.

Time-varying signals transmission tests

Taking into account the above analysis, in order to ensure low nonlinear distortions during the transmission of time-varying signals through the OLED(SY)/OLSD(IPT) optocoupler, the operating point determined by the input voltage $u_{in,dc}$ = 7 V (current density $j_{in,dc}$ = 200 mAcm^−2^) was chosen. The corresponding constant component of the radiation emittance was $m_{dc}$ = 16 Wm^−2^. The variable component consisted of sinusoidal voltage waves $u_{in\sim }\left(t\right)=u_{in0}\sin\left(2\pi ft\right)$ and rectangular $u_{in⊓}\left(t\right)=u_{in0}\left[1\left(t\right)-1\left(t-0.5/f\right)+1\left(t-1/f\right)-t-1.5/f+\cdots .\right]$, where *t* is time, *f* is frequency, and 1(*t*) is the Heaviside step function. The amplitude of the variable component $u_{in0}$ was 0.1 V. It corresponded to the range of transmitter current changes from 190 to 210 mAcm^−2^. For the adopted operating point and the range of transmitter current variation, both the emitter radiative emittance values and the corresponding photodetector photocurrent were within the ranges of linear operation of the OPC (see the inset in Figure 5).

**Figure 8:**
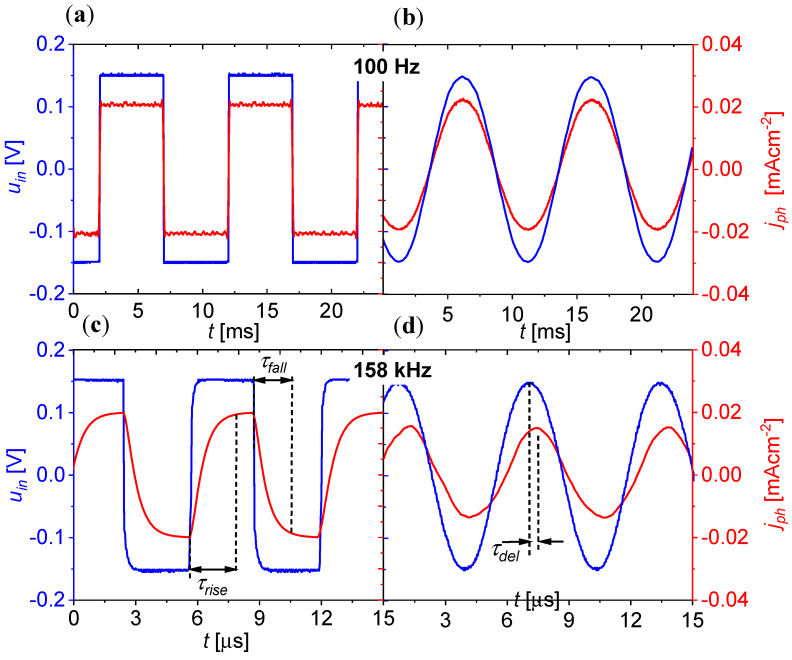
Variable components of current density at the output of the OLED/OLSD optocoupler $j_{ph⊓}$ and $j_{ph\sim }$ as a function of time t (red) after applying a voltage wave (blue) to the transmitter input. (**a**,**c**) Rectangular $u_{in⊓}$ and (**b**,**d**) sinusoidal $u_{in\sim }$ of two frequencies of 100 Hz and 158 kHz; (**c**) the symbols $\tau_{rise}$ and $\tau_{fall}$ denote the times of the rise and fall of the current at the output, respectively, after applying a rectangular voltage wave to the input; (**d**) $\tau_{del}$ is the delay time of the receiver photocurrent after applying a voltage harmonic component to the input.

Figure 8 shows sample waveforms of the variable components of the voltage applied to the device input $u_{in⊓}$ and $u_{in\sim }$ and the corresponding current densities at the photodetector output $j_{ph⊓}$ and $j_{ph\sim }$ developed based on the measured oscillograms (see Supplementary Information Figure S5). The results are presented for two selected frequency values of 100 Hz and 158 kHz. At 100 Hz, there was no attenuation of the signal at the output of the device. There was no phase shift in the output current relative to the input voltage, and the input current increased immediately after the voltage jump (on a time scale corresponding to the period of a rectangular voltage wave). However, at a frequency of 158 kHz, the response to a rectangular wave did not resemble the shape of the input voltage. The rise and fall of the output current corresponded to the rise ($\tau_{rise}$) and relaxation ($\tau_{fall}$) times of 2 μs, respectively.

After the deconvolution of the waveform of the variable component shown in the oscillogram in Figure S5, we obtained two time constants $\tau_{1}$ = 249 ns and $\tau_{2}$ = 440 ns. For the former, the three-decibel cutoff frequency ($1/\left(2\pi \tau_{1}\right)$ was 660 kHz, which roughly corresponded to the transmitter cutoff frequency $f_{-3dB,OLED}$. The second time constant $\tau_{2}$ indicated that the three-decibel receiver cutoff frequency should be about 360 kHz, which is higher than $f_{-3dB,OLSD}$ = 250 kHz for OLSD(IPT) (see Supplementary Information, Table S1).

**Figure 9:**
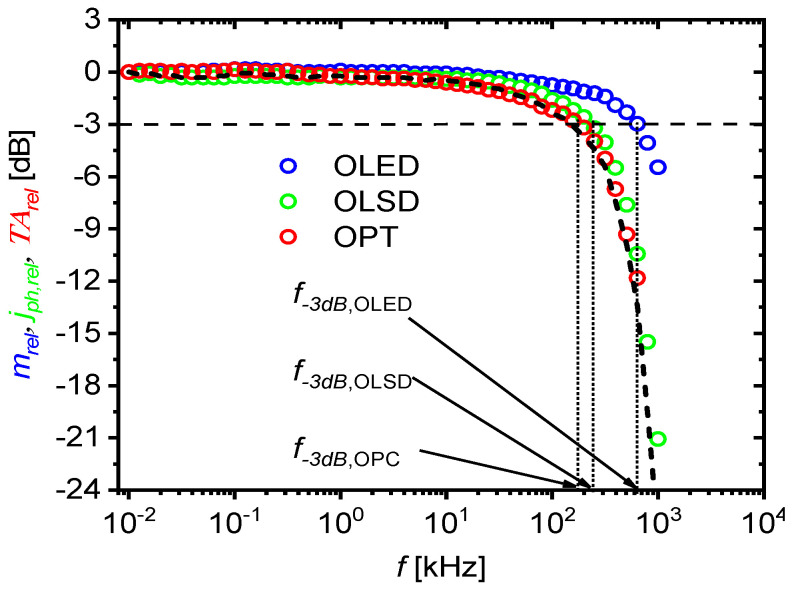
Comparison of frequency spectra for relative amplitude of radiant emittance $m_{rel}$ of the OLED (blue circles), relative amplitude of photocurrent density $j_{ph,rel}$ of the OLSD photodiode (red circles), and relative transadmittance ${TA}_{rel}$ of the OLED/OLSD optocoupler (green circles). Dashed curve—spectrum resulting from multiplication of $m_{rel}$ and $j_{ph,rel}$ spectra. Vertical dotted lines indicate three-decibel cutoff frequencies of OLED $f_{-3dB,SY}$*,* OLSD $f_{-3dB,IPT}$ and OPC $f_{-3dB,OPT}$.

For a sinusoidal signal with a frequency of 158 kHz, the output current amplitude decreased by $\sqrt{2}$ compared to the amplitude for lower frequencies (100 Hz). This would indicate that 158 kHz is equal to the three-decibel cutoff frequency $f_{-3dB,OPC}$, but the phase shift between the input voltage and the output current was less than π/4 ($\tau_{rise}=1/\left(8f\right)$). This in turn would mean that the three-decibel cutoff frequency was higher than 158 kHz. In order to determine $f_{-3dB,OPC}$, the frequency dependence of the relative transadmittance ${TA}_{rel}(f)$ was investigated, calculated as the quotient of the amplitudes of the variable components of the current at the photodetector output, and the voltage applied to the transmitter input for frequency *f* was studied by referring to the same quotient, but for the undamped frequency of 10 Hz: ${TA}_{rel}\left(f\right)=\left[i_{in0}\left(f\right)u_{in0}\left(10Hz\right)\right]/\left[u_{in0}\left(f\right)i_{in0}\left(10Hz\right)\right]$. Figure 9 shows a comparison of the frequency spectra for the relative amplitude of radiant emittance ($m_{rel}\left(f\right)=m_{0}\left(f\right)/m_{0}\left(10Hz\right)$) for OLED, relative amplitude of photocurrent density $j_{ph,rel}\left(f\right)=j_{ph,0}\left(f\right)/j_{ph,0}\left(10Hz\right)$ recorded by OLSD, product $m_{rel}\left(f\right)j_{ph,rel}\left(f\right),$ and relative transadmittance ${TA}_{rel}\left(f\right)$. As expected, the spectrum obtained by multiplying the transmitter and receiver spectra ($m_{rel}\left(f\right)j_{ph,rel}\left(f\right)$) completely overlapped with the relative transadmittance spectrum of the optocoupler in a wide range of considered frequencies (from 10 Hz to 640 kHz). The determined three-decibel cutoff frequency of the OLED/OLSD optocoupler was 170 kHz.

The spectrum obtained by mathematically combining the frequency spectra of the transmitter and photodetector matched the transadmittance spectrum measured for the optocoupler almost perfectly. Considering the considerable t time interval (several weeks) between the time of conducting a complete set of experiments, first performed separately for the transmitter and photodetector, and then the production of the optocoupler and its characterization, the overlap of the spectra indicated the stability of the fabricated devices. It could be expected that applying a reverse voltage to the photodetector would increase the cutoff frequency of the device. This broadening of the bandwidth also follows from the analysis of the OPC equivalent circuit (see Figure S3 in Supplementary Information), where capacitor $C_{re}$ symbolizes the photodiode capacitance, which is the resultant of the geometric capacitance, the capacitance of the electrode–semiconductor junctions, and the capacitance of the bulk heterojunction. The application of an external electric field causes a decrease in junction capacitance [48,49,50] and this in turn causes an increase in limit frequency $f_{-3dB,OPC}$. As a result, the frequency band for signals that can be transmitted without distortion is extended. However, for the OLED/OLSD optocouplers tested, no effect of increasing the limit frequency $f_{-3dB,OPC}$ was observed after applying a reverse voltage to the photodetector. This may be explained by the low leakage resistance and the small number of junctions in the simple structure of the OLSD photodetector tested compared to typical multilayer photodiodes described in the literature. In these photodiodes, the leakage current reduction is achieved by introducing additional interlayers [14,41] to block the injection of holes from the cathode and electrons from the anode. However, the additional junctions created by the interlayers hinder the charge carrier transport for alternating signals, because charges are accumulated near these junctions. In order to accelerate the operation of the device, these space-charges should be removed, which is achieved by applying a reverse voltage to the photodiode. In the case of our single-layer OLSD, the charges from the active layer and the areas near the junctions are quickly removed even with shorted electrodes due to the low leakage resistance; in this case, the application of a reverse voltage does not improve the photodiode dynamics.

It should be emphasized that it is difficult to produce an optocoupler that has both a high *CTR* and fast response time. In general, it can be seen that optocouplers with high *CTR* values tend to operate slower than optocouplers with low *CTR* [8,41,51,52,53]. The improvement in receiver speed is usually achieved by biasing the OPDs with the voltage $u_{rev}$. The assumption is that an increase in the effective electric field leads to less carrier trapping and higher effective mobility, resulting in faster carrier extraction from the active layer. However, in our devices, we obtained a high value of $f_{-3dB,OPC}\;$= 170 kHz, comparable to other OPC devices fabricated using similar organic semiconductors, but without the need to apply $u_{rev}$. To the best of our knowledge, the three-decibel frequency for the photodetectors with an ITIC:PTB7-Th mixture layer has not yet been determined. The published data for OPD with different mixtures of the PTB7-Th/acceptor [29,30] or with ITIC/donor [14,32] show that only photodetectors with the mixture PTB7-Th:CO1-4Cl had cutoff frequencies $f_{-3dB,OPC}\;$= 240 kHz comparable to our results [54,55].

The results presented in this article allow us to formulate several detailed summary statements. An organic light-emitting diode with a single active layer made of the well-known Super Yellow can be used as an efficient transmitter. An organic light-sensitive diode with a single active layer made of a mixture of a polymer donor PTB7-Th and a low-molecular-weight non-fullerene acceptor ITIC can be used as an efficient receiver. An OPC can be easily formed by inducing a tight adhesion of the glass substrates of OLED and OLSD, since the OLED and OLSD have the same geometry and structure: glass/ITO/PEDOT:PSS/(active layer)/Ca/Al. Despite their simplified structure, our OPC devices showed the ability to transmit current signals over a wide frequency band with a good linearity of current transmission. The current transfer ratio (CTR) was 0.13%; the three-decibel cutoff frequency was 170 kHz for harmonic signals, and the response time to a step change in the current at the OPC input was 2 μs.

## 4. Conclusions

- We have shown that it is possible to produce an optocoupler that meets the application requirements using commercially available organic semiconductors that can be processed using solution techniques.
- The organic light-sensitive diode can efficiently operate as a receiver with short-circuited electrodes, i.e., without any reverse voltage applied. It is often stated in the literature that it is necessary to apply a reverse voltage to the photodiode to make it operate in the third quadrant of its I-V characteristic and to extend the frequency bandwidth of the device. However, from our analysis, it is clear that in order to minimize linearity degradation, the photodetector should not be biased or should be biased with the lowest possible reverse voltage.
- Compared to most OPC devices with complex multi-layer transmitter and receiver structures and the need to apply reverse voltage to the receiver, the simplest possible design of our OPC reduces the number of manufacturing steps and greatly simplifies the device manufacturing process.
- The simple structure of our OPC, if the vacuum-deposited Ca/Al electrodes are replaced with a conductive ink (e.g., zinc oxide and nanosilver suspensions), enables the production of optocouplers by printing on flexible double-sided ITO-coated substrates. This structure will also reduce light losses resulting from reflections at interfaces.

## Data Availability

The original contributions presented in this study are included in the article/Supplementary Material. Further inquiries can be directed to the corresponding author.

## Supplementary Materials

The following supporting information can be downloaded at https://www.mdpi.com/article/10.3390/ma18010152/s1.
