# Supplementary material for: Organic Optocoupler with Simple Construction as an Effective Linear Current Transceiver
Source: Materials (Basel). 2025 Jan 2;18(1):152. doi: 10.3390/ma18010152 (PMC11722434; doi:10.3390/ma18010152)
Supplement: Supplementary file 1 [file materials-18-00152-s001.zip › materials-3386038-supplementary.pdf]

Organic optocoupler with simple construction as an effective linear current transceiver

Supplementary information

Jaroslaw Jung[*1], Arkadiusz Selerowicz[1], Jacek Ulanski[1], Ruslana Udovytska[1], Beata Luszczynska[1], Artur Zawadzki[2], Andrzej Rybak[2]

[1]Department of Molecular Physics, Faculty of Chemistry, Lodz University of Technology, 116 Zeromskiego Str., 90-924 Lodz, Poland,
[2] ABB Corporate Technology Center, 13A Starowislna Str., 31-038 Krakow, Poland.

Supplement S1 ***Multilayer optocoupler (OPC) structure and circuit diagram for testing electrical properties of OPC***

Supplement S2 - Electrical equivalent circuit of the optocoupler containing peripheral components

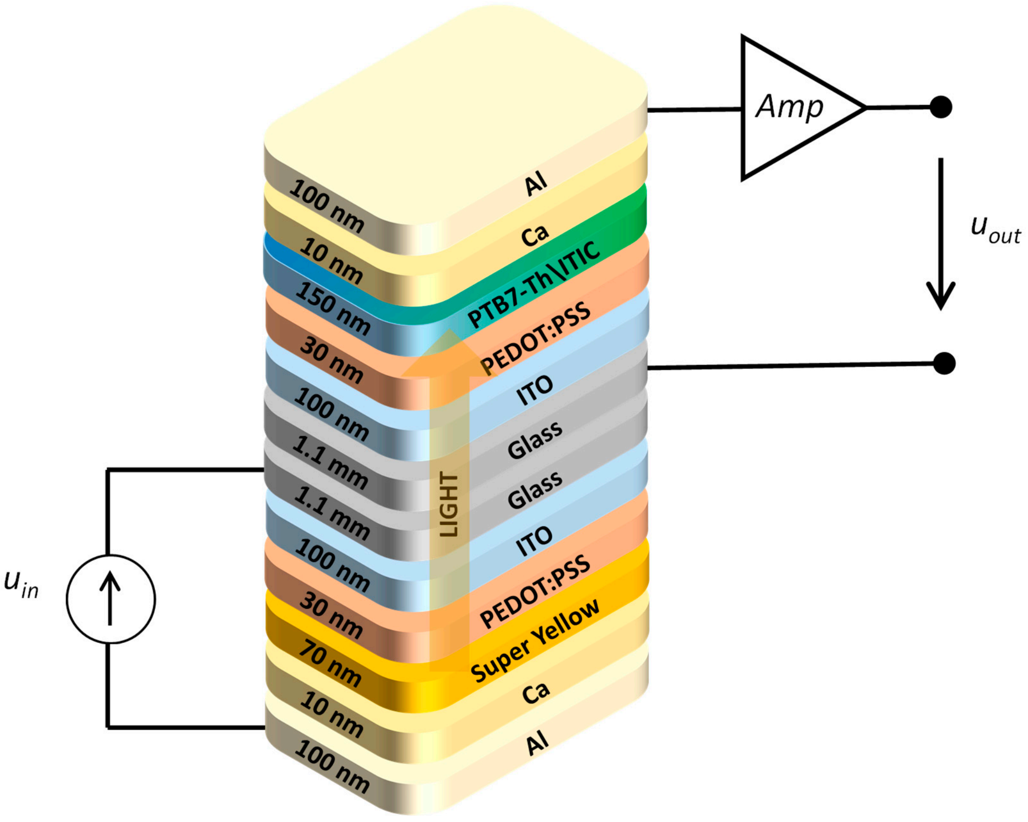

Figure S1 Scheme of the layer structure of the optocoupler and the thicknesses of the individual layers

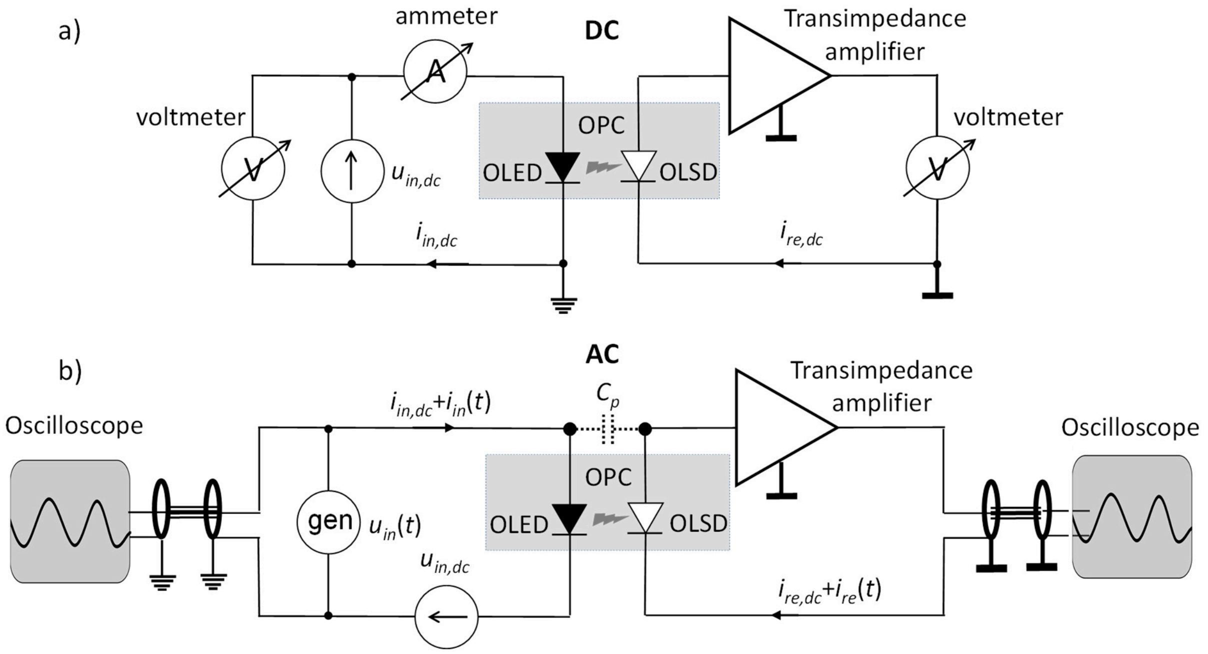

Figure S2 Scheme of electric circuits for the (a) DC and (b) AC working regimes of OPC; the symbols represent: $u_{in,dc}$ emitter (OLED) input DC bias voltage, $i_{in,dc}$ - emitter input DC current, $u_{in}(t)$ - emitter input AC voltage, $i_{in}(t)$ – emitter input AC current, $i_{re,dc}$- receiver (OLSD) output DC current, $i_{re}(t)$ - receiver output AC current; C_p - equivalent parasitic capacitance responsible for crosstalk between the OPC input and output circuits.

Below is presented an analysis of the equivalent circuit diagram of the optocoupler transmitter and receiver circuits shown in Fig. S3, in which a direct current of intensity $i_{in,dc}$ is applied to the input and a harmonic alternating current $i_{in}(t)$ is added to it with an amplitude i_{in0} much smaller than $i_{in,dc}$. It is assumed that the dynamic resistance of the transmitter ($R_{diff,em}$ - the tangent of the slope angle of the current-voltage characteristic of the emitter) in the vicinity of the operating point is constant and that the noise occurring in the transmitter and receiver can be neglected.

The total current in the node (n_{em}) of the emitter circuit is as follows:

$$i_{in,dc} + i_{in}(t) = i_{em,dc} + i_{em}(t) + i_{em,C}(t), \tag{S1}$$

where $i_{em}(t)$ is the emitter current and $i_{em,C}(t)$ is the charging current of the capacitor (C_{em}) with capacitance equal to the sum of the geometric capacitance and the capacitance of the emitter junctions, and are given by:

$$\begin{aligned} i_{em}(t) &= \frac{u_{em,C}(t)}{R_{diff,em}} \\ i_{em,C}(t) &= C_{em}\frac{du_{em,C}(t)}{dt} \end{aligned} \tag{S2}$$

where $u_{em,C}(t)$ is the voltage drop across the capacitor C_{em}.

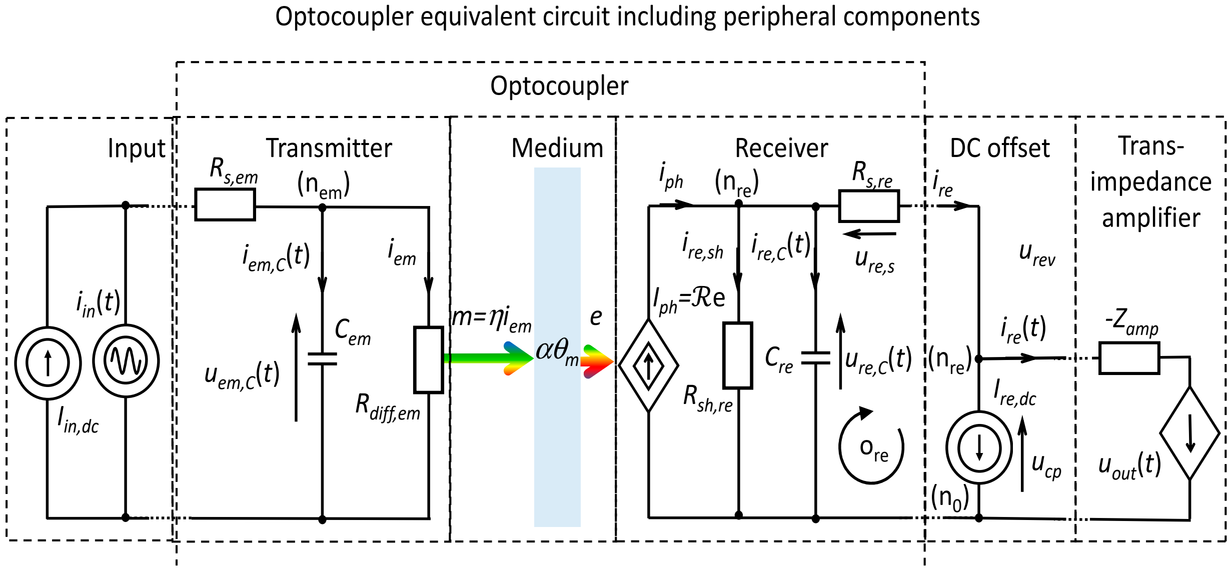

Figure S3. Equivalent electrical circuit of an optocoupler containing a transmitter and receiver, a DC compensation circuit, and a transimpedance amplifier. The optocoupler is controlled from external DC and AC sources $i_{in,dc}$ and $i_{in}(t)$, respectively. The transmitter elements are, respectively, the contact resistance R_{diff}, the capacitance C_{em}, and the dynamic resistance R_{diff} whose value depends on the selected operating point. The transmitter emits light of radiant emittance m with current efficiency η. The light, after passing through a medium with the ability to concentrate light α and transmit ϑ_m, irradiates the photodetector with intensity e. In the receiver, the photodetector converts light into a photocurrent with strength equal to the product of the irradiation and the photodetector responsivity $\mathcal{R}$. The photodetector elements are, respectively, the contact resistance $R_{s,re}$, the leakage resistance $R_{sh,re}$ and the capacitance C_{re}. An external current source with the efficiency $i_{re,dc}$ is used to eliminate the DC component of the photocurrent. The amplifier with the transimpedance $-Z_{amp}$. converts the variable component of the receiver current $\mathrm{i_{re}}(t)$ into the voltage $u_{out}(t) = -Z_{amp} i_{re}(t)$.

The alternating component $i_{in}(t)$ of the input current is given by the function:

$$i_{in}(t) = i_{in0} sin(2\pi f t) \tag{S3}$$

where f is the frequency.

In the steady state, solving the system of equations (S1) and (S2) gives expressions for the alternating ($i_{em}(t)$) components of the emitter current, the amplitude (i_{em0}) of the alternating current, and the three-decibel cut-off frequency ($f_{-3dB,em}$):

$$\begin{aligned} i_{em}(t) &= i_{em0} sin\left(2\pi f t - arctg\left(\frac{f}{f_{-3dB,em}}\right)\right) \\ i_{em0} &= i_{in0}\left(1 + \frac{f^2}{f_{-3dB,em}^2}\right)^{-1/2} \\ f_{-3dB,em} &= \frac{1}{2\pi}\left(R_{diff,em} C_{em}\right)^{-1} \end{aligned} \tag{S4}$$

The flow of the alternating current component $i_{em}(t)$ causes the emission of an alternating light with the amplitude of the spectral emittance ($m_{0,\lambda,f}$). Similarly to the amplitude of the current i_{em0}, $m_{0,\lambda,f}$ is attenuated at frequencies higher than $f_{-3dB,em}$:

$$m_{0,\lambda,f} = m_{0,\lambda}\left(1+\frac{f^2}{f_{-3dB,em}^2}\right)^{-1/2} \quad \text{(S5)}$$

where $m_{0,\lambda}$, is the amplitude of the spectral emittance for frequencies much lower than $f_{-3dB,em}$.

The total spectral emittance has two components, constant (m_{dc}) and variable ($m(t)$). Through a medium (e.g. glass) with spectral transmittance $\theta_m(\lambda)$ only part of the emitted light reaches the photodetector surface (in many real devices, an additional optical system with a light concentration coefficient on the photodetector surface α is placed between the transmitter and the receiver, intended to collimate the light beam - in our case $\alpha\theta_m(\lambda) = 1$). The total irradiance (its constant (e_{dc}) and variable ($e(t)$) components) is:

$$e_{dc} + e(t) = \int_{\lambda_1}^{\lambda_2} \alpha\theta_m(\lambda)\left[m_{dc,\lambda} + m_\lambda(t)\right]d\lambda \quad \text{(S6)}$$

where λ_1 and λ_2 are the wavelengths of light equal to the lowest and highest wavelength of the absorbed light.

The efficiency of the current source (I) in the photodetector is proportional to the total irradiance and is therefore equal to:

$$I = I_{dc} + I_0\left(1+\frac{f^2}{f_{-3dB,em}^2}\right)^{-1/2} sin\left(2\pi ft - arctg\left(\frac{f}{f_{-3dB,em}}\right)\right) \quad \text{(S7)}$$

where I_{dc} is the constant component of the efficiency of current source and I_{ph0} is the amplitude of the variable component of the efficiency of current source ($I(t)$).

The total current and voltage, respectively, in the node (n_{re}) and loop (o_{re}) of the receiver circuit are as follows:

$$\begin{aligned} &i_{ph,dc} + i_{ph}(t) = i_{re,dc} + i_{re}(t) + i_{re,sh,dc} + i_{re,sh}(t) + i_{re,C}(t) \\ &u_{re,s} + u_{re,s}(t) = u_{re,C}(t) + u_{cp} \end{aligned} \quad \text{(S8)}$$

where $i_{ph,dc} = I_{dc}$ and $i_{ph}(t) = I(t)$ are the constant and variable components of the photocurrent, respectively; $i_{re,dc}$ and $i_{re}(t)$ are the constant and variable components of the current at the receiver output; $i_{re,sh,dc}$ and $i_{re,sh}(t)$ are the constant and variable components of the receiver leakage current through the shunt resistance ($R_{sh,re}$), respectively; $i_{re,C}(t)$ is the charging current of the capacitor symbolizing the electric capacitance of the receiver (C_{re}); $u_{re,s}$ and $u_{re,s}(t)$ are the constant and variable components of the voltage drop across the contact resistance ($R_{s,re}$) respectively; $u_{re,C}(t)$ is the voltage drop across the capacitor C_{re}, and u_{cp} is the voltage drop across the current source in the correction circuit; where the following relationships hold:

$$\begin{aligned} &i_{re,sh}(t) = \frac{u_{re,C}(t)}{R_{sh,re}} \\ &i_{re,C}(t) = C_{re}\frac{du_{re,C}(t)}{dt} \\ &u_{re,s} = i_{re,dc}R_{s,re} \\ &u_{re,s}(t) = i_{re}(t)R_{s,re} \\ &u_{cp} = 0\ \mathrm{V} \end{aligned} \quad \text{(S9)}$$

Solving the system of equations (S7), (S8) and (S9) gives expressions for: the current components $i_{re,dc}$ and $i_{re}(t)$, the amplitude (i_{re0}) of the alternating current and the three-decibel cut-off frequency ($f_{-3dB,re}$):

$$\begin{aligned}
&\text{a)}\quad i_{re,dc} = \frac{R_{sh,re}}{R_{sh,re}+R_{s,re}} i_{ph,dc}\\
&\text{b)}\quad i_{re}(t) = i_{re0} sin\left(2\pi f t - arctg\left(\frac{f}{f_{-3dB,re}}\right) - arctg\left(\frac{f}{f_{-3dB,em}}\right)\right)\\
&\text{c)}\quad i_{re0} = \frac{R_{sh,re}}{R_{sh,re}+R_{s,re}} i_{ph0}\left(1+\frac{f^2}{f_{-3dB,em}^2}\right)^{-1/2}\left(1+\frac{f^2}{f_{-3dB,re}^2}\right)^{-1/2}\\
&\text{d)}\quad f_{-3dB,re} = \frac{1}{2\pi}\left(\frac{R_{s,re}R_{sh,re}}{R_{sh,re}+R_{s,re}} C_{re}\right)^{-1}
\end{aligned} \tag{S10}$$

According to the adopted equivalent model, the three-decibel limit frequency of the optocoupler ($f_{-3dB,OPC}$) is a solution to the quadratic equation that arises after equating the expression under the root in the formula (S10) to the number 2, and it is equal to:

$$f_{-3dB,OPC} = \sqrt{\frac{1}{2}\left(\sqrt{\left(f_{-3dB,re}^2 + f_{-3dB,em}^2\right)^2 + 4 f_{-3dB,re}^2 f_{-3dB,em}^2} - f_{-3dB,re}^2 - f_{-3dB,em}^2\right)} \tag{S11}$$

It should be noted that the highest three-decibel frequency value $f_{-3dB,OPC}$ will always be smaller than the smaller of the both frequencies $f_{-3dB,em}$ and $f_{-3dB,re}$, and in the case when they are equal ($f_{-3dB,em} = f_{-3dB,re} = f_{-3dB}$) then $f_{-3dB,OPC} \cong 0.64 \cdot f_{-3dB}$.

Supplement S3 - OLSD AC parameters

OLSDs had active layers with donor-acceptor bulk heterojunction. The acceptor component was either an ITIC or a macromolecular semiconductor Poli{{[N,N'-bis(2-oktyldodecylo)naftaleno-1,4,5,8-bis(dikarboksyimid)-2,6-diylo]-alt-5,5'-(2,2'-bitiofen)}-ran-{[N,N'-bis(2-oktyldodecylo)naftaleno-1,4,5,8-bis(dikarboksyimid)-2,6-diylo]-alt-2,5-tiofen}} (PNDI-T10). On the other hand, the donor was PTB7-Th or Poli[4,8-bis(5-(2-etyloheksylo)tiofen-2-ylo)benzo[1,2-b;4,5-b']ditiofeno-2,6-diyl-alt-5,5-(1',3'-di-2-tienylo-5',7'-bis(2-etyloheksylo)benzo[1',2'-c:4',5'-c']ditiofeno-4,8-dion] (PBDB-T). Table ST1 summarizes the AC parameters of five OLSDs that were tested as receivers in optocouplers with SY-based OLEDs. The abbreviations mean: IPT - a mixture of ITIC with PTB7-Th; PPB - a mixture of PNDI-T10 with PBDB-T and PPT - a mixture of PNDI-T10 with PTB7-Th. The numbers separated by a colon are the weight proportions of the mixture components, and the abbreviations chb and chf denote the solvent from which the layers were obtained: chf - chloroform and chb - chlorobenzene.

Table ST1

	IPT1:1chb	IPT1:1chf	IPT3:2chf	PPB3:2chf	PPT3:2chf
$\tau_{rise/fall}$ [μs]	2,0	5,6	3,2	3,4	2,0

$f_{-3dB,OPC}$ [kHz]	250	110	214	250	330

Figure S4a shows a comparison of the spectral emittance $m(\lambda)$ for SY-based OLED, measured at a supply voltage of 7 V, with the responsivity spectra $\mathcal{R}(\lambda)$ for OLSDs. The product of these spectra is shown in Figure S4b. The largest possible photocurrent that the photodetector could measure is equal to the integral of the resultant spectrum $m(\lambda) \cdot \mathcal{R}(\lambda)$.

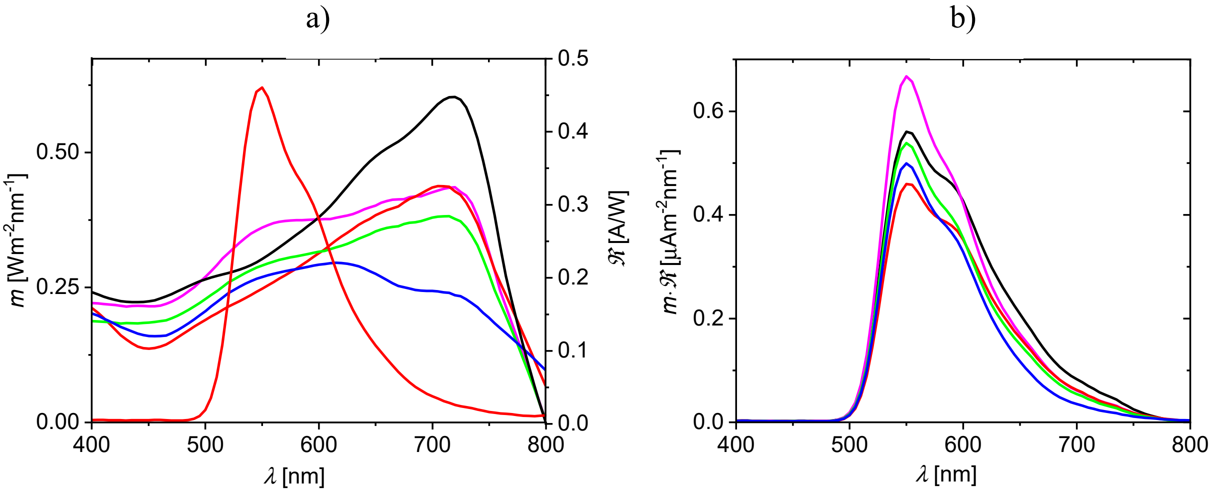

Figure S4 a) The spectral emittance $m(\lambda)$ of the SY-based OLED transmitter and the responsivity spectra $\mathcal{R}(\lambda)$ of the OLSDs; b) the product of the spectra $m(\lambda) \cdot \mathcal{R}(\lambda)$.

Supplement S4 - Deconvolution of photocurrent induced by a voltage step at the input of an optoisolator

The graphs shown in Figures 8a,b,d were obtained based on smoothed oscillograms, while the photocurrent waveform shown in Figure 8c was obtained by fitting the model to the curve visible in the oscillogram (Figure S5) and then removing the current spikes. This signal appeared in the measurement system both when the optocoupler holder was empty and when a working optoisolator was installed.

The voltage spikes visible on the oscilloscope screen were caused by the presence of parasitic capacitance C_p between the transmitter and receiver circuits (see Figure S2).

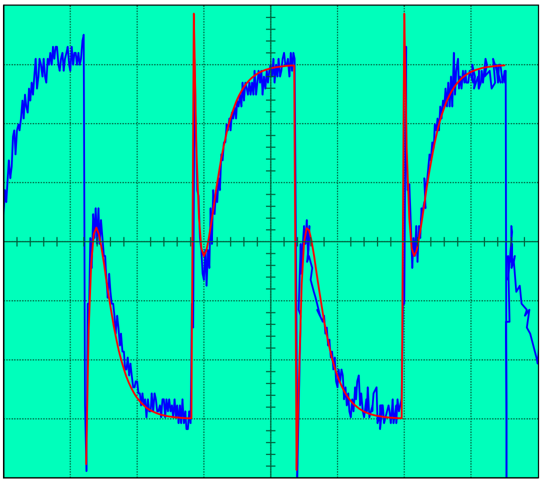

Figure S5 Oscillogram showing the time dependence of the variable voltage component at the output of a transimpedance amplifier connected to the output of an optocoupler. A square voltage wave with an amplitude of 0.1 V and a frequency of 158 kHz was applied to the OPC input.

Let's analyze the total current that flowed into the transimpedance amplifier during the measurements. The variable component of the current at the amplifier input was:

$$i_{amp} = i_{re}(t) + i_p(t) \tag{S12}$$

where i_p was the charging current of the capacitor with capacitance C_p. It was assumed that after applying a square wave of current to the input of the optocoupler, the dependence of the current i_p on time could be approximated as follows:

$$i_p(t) = i_{p0}e^{-t/\tau_p} \tag{S13}$$

where i_{p0} is the discharge current at time t = 0 s, and τ_p is the discharge time constant C_p.

Moreover, it was assumed that the variable component of the photocurrent in the receiver was delayed due to the existence of time constants τ_{em} and τ_{re} characteristic of the transmitter and receiver circuits (approximately, the relations $\tau_{em} = 1/\left(2\pi f_{-3dB,em}\right)$ and $\tau_{em} = 1/\left(2\pi f_{-3dB,re}\right)$ should hold).

The sum of the variable components of the currents at a node (n_{em}) in the emitter equivalent circuit is as follows (see Figure S3):

$$i_{in}(t) = i_{em}(t) + C_{em}R_{diff,em}\frac{di_{em}(t)}{dt} \tag{S14}$$

The two-sided Laplace transform of the equation (S14) for a step change in current at the input of the optocoupler gives the equation:

$$\frac{i_{in,m}}{s} = I_{em}(s) + s\tau_{em}I_{em}(s) \tag{S15}$$

where s is the Laplace variable, $i_{in,m}$ is the amplitude of the current step at the transmitter input, $\tau_{em} = C_{em}R_{diff,em}$ is the time constant of the emitter circuit, and $I_{em}(s)$ is the Laplace transform of the emitter current.

After making the transformations, one gets:

$$I_{em}(s) = i_{in,m}\frac{1}{s(1+s\tau_{em})} \tag{S16}$$

The sum of the variable components of the currents at a node (n_{re}) is as follows (see Figure S2):

$$i_{ph}(t) = i_{re}(t) + \frac{R_{s,re}}{R_{sh,re}}i_{re}(t) + R_{s,re}C_{re}\frac{di_{re}(t)}{dt} \tag{S17}$$

After the Laplace transformation of the equation (S17) and after subsequent transformations, the Laplace transform of the receiver current ($I_{re}(s)$) is:

$$I_{re}(s) = I_{ph}(s)\frac{R_{sh,re}}{R_{sh,re}+R_{s,re}}\frac{1}{s(1+s\tau_{re})} \tag{S18}$$

where $\tau_{re} = \frac{R_{sh,re}R_{s,re}}{R_{sh,re}+R_{s,re}}C_{re}$ is the time constant of the receiver circuit and $I_{em}(s)$ is the Laplace transform of the emitter current.

Since $I_{ph}(s) = CTRI_{em}(s)$, after taking into account the formulas (S16) and (S18) we get:

$$I_{re}(s) = i_{ph,m}\frac{1}{s(1+s\tau_{em})(1+s\tau_{re})} \tag{S19}$$

where $i_{ph,m} = i_{in,m}CTR\frac{R_{sh,re}}{R_{sh,re}+R_{s,re}}$ is the amplitude of the photocurrent jump in the receiver.

The total current in the node (o_{re}) of the receiver circuit is equal to the sum of the receiver currents and the parasitic current. After performing the inverse Laplace transform of the equation (S19), taking into account the equation (S13) and the transconductance in the transimpedance amplifier coupling system ($-G_{amp}$), we get the equation for the time dependence of the voltage (u_{amp}) at the amplifier output:

$$u_{amp}(t) = \left\{i_{ph,0}\left[1 - \frac{1}{\tau_{em}-\tau_{re}}\left(\tau_1 e^{-t/\tau_{em}} - \tau_{re}e^{-t/\tau_{re}}\right)\right] + i_{p0}e^{-t/\tau_p}\right\}G_{amp}{}^{-1} \tag{S20}$$

For the amplifier transconductance $-G_{amp} = 10^{-5}$ S, the curve described by the equation (S20) was fitted to the measured time dependencies $u_{amp}(t)$ visible in the oscillogram in Figure S5 (for the falling current, the right side of the equation with a minus sign was assumed).

The following results were obtained:

The amplitude of the parasitic capacitance charging current, the source of which was the generator at the OPC input, was i_{p0} = 760 nA. This was only 0.16% of the current supplied from the generator to the OPC input. The time constant of charging the parasitic capacitors was τ_p = 160 ns.

The observed value of the photocurrent amplitude was $i_{ph,0}$ = 1.2 μA The time constant τ_{em}= 240 ns corresponded well with the time constant determined from the measured three-decibel frequency of the OLED transmitter ($1/\left(2\pi f_{-3dB,em}\right)$ = 250 ns). On the other hand, the time constant τ_{re} = 440 ns lower smaller than the time constant determined for the OLSD:IPT1:1chb receiver based on the three-decibel frequency ($1/\left(2\pi f_{-3dB,em}\right)$ = 630 ns (see Table ST1).
